# *LMNA*-associated myopathies: the Italian experience in a large cohort of patients

**DOI:** 10.1186/1750-1172-10-S2-O19

**Published:** 2015-11-11

**Authors:** Lorenzo Maggi

**Affiliations:** 1 Neuromuscular Diseases and Neuroimmunology Unit, Fondazione IRCCS Istituto Neurologico Carlo Besta, Milan, Italy

## 

We conducted a retrospective study in a large cohort of myopathic patients carrying *LMNA* gene mutations to evaluate clinical and molecular features associated with different phenotypes. To this purpose we included 90 *LMNA*-mutated myopathic patients and 36 *LMNA*-mutated familial cases without muscle involvement. Among the myopathic patients LGMD1B was by far the most frequent phenotype, observed in 43 (48%) patients, followed by L-CMD in 21 (23%), EDMD2 in 20 (22%) and an atypical myopathy in 6 (7%). The different myopathic phenotypes shared a similar cardiac impairment. On the other hand comparing cardiac features between myopathic and familial cases without muscle involvement we observed that cardioverter defibrillator or pacemaker were implanted more frequently in myopathic patients (n=43) (*p*=0.006). In addition heart transplantation and death were observed only in myopathic subgroup, respectively in 8 (9%) and 10 (11%) patients. In conclusion *LMNA*-related myopathies represent a continuum clinical spectrum; their clinical course appears to be dominated by cardiac involvement, considering the relatively low frequency of other complications, including loss of ambulation, assisted ventilation, surgery for scoliosis or gastrostomy. Longitudinal studied are needed to better investigate their natural history and provide indications for early management of heart involvement, in particular in first decades of life, to prevent the risk of fatal events.

